# Teleneurorehabilitation program (virtual reality) for patients with balance disorders: descriptive study

**DOI:** 10.1186/s13102-021-00314-z

**Published:** 2021-08-02

**Authors:** Marcos Maldonado-Díaz, Patricia Vargas, Ricardo Vasquez, Felipe Gonzalez-Seguel, Betel Rivero, Viviane Hidalgo-Cabalín, Tania Gutierrez-Panchana

**Affiliations:** Clinica Alemana Universidad del Desarrollo, Vitacura 5951. Región Metropolitana, Santiago, Chile

**Keywords:** Telerehabilitation, Virtual reality, Balance, Reliability, Clinical outcomes

## Abstract

**Background:**

Balance disorders are common in patients with neurological or vestibular diseases. Telerehabilitation program is a treatment to be as safe as conventional treatment. One of the most used methods to perform telerehabilitation is the incorporation of Virtual Reality. In general, rehabilitation programs train predictive postural control, so the patient does not always acquire the necessary autonomy to react to situations of instability. On the other hand, the objective and systematic supervision and measurement of these programs is limited, making it necessary to create clinical protocols with precise and measurable rehabilitation objectives. This study present the training selection methodology and clinical protocol for patients with balance disorders inserted in a Telerehabilitation Program based on Virtual Reality.

**Methods:**

Descriptive study where physiotherapists were trained to use RehaMetrics®. To evaluate their level of agreement in the selection of the exercise clusters developed, the Interobserver Reliability was measured through the kappa statistic. Subsequently, the exercises were applied to a group of patients recruited with sedentary trunk control (Berg Balance Scale = 3 points in item 3), mild or normal cognitive level (Montreal Cognitive Assessment> 21 points), and prescribed for tele-rehabilitation by a doctor.

**Results:**

The agreement among the expert physiotherapists irrespective of the cluster exceeds 80%, which indicates a very good strength of agreement, while the novices reached a level of agreement of 45%, which suggests a moderate strength of agreement. All clinical outcomes showed statistically significant differences between the median times, as did the Maximum Width Left Side (MWLS) (cm). The average number of minutes of training was 485.81 (SD 246.49 min), and the number of sessions performed during the 4 weeks of intervention was 17 (SD 7.15 sessions).

**Conclusions:**

This analysis what had excellent interobserver reliability with trained physiotherapists. Regarding the second phase of the study, the results show a statistically significant difference between the initial and final evaluation of the clinical tests, which could result in better performance in aspects such as: balance, gait functionality, meter walked and cognition. Telerehabilitation Program based on Virtual Reality is an excellent alternative to provide continuity of treatment to patients with balance disorders.

**Supplementary Information:**

The online version contains supplementary material available at 10.1186/s13102-021-00314-z.

## Background

A key factor that ultimately determines whether an individual will fall is the ability to react to a loss of balance. This ability is called reactive balance control. This ability requires that a postural response is executed with sufficient speed and strength. However, latency times is correlated with age [1, 2]. In fact, older adults show greater variability in reactive postural responses than younger adults, also if they are also developing a peripheral vestibular disease, the response worsens [3, 4]. Even more, impaired reactive balance control predicts increased risk of falls after discharge following inpatient stroke rehabilitation [5–7].

Maintaining balance stability in dynamic motion requires adequate recovery mechanisms and strategies to handle the sudden shift of the center of mass displacement [2, 8]. While traditional balance training, focused on maintaining stability during voluntary movement, prevents falls in older adults [9–11], these programs do not reduce fall rates in individuals with stroke and vestibular diseases. Reactive postural control training focuses on practicing responses to instability, with the goal of improving reactive balance control and reducing the risk of falls [4, 12].

According to the principles of motor learning, balance training requires it to be repetitive, intensive and task-oriented exercises [13]. Many patients choose to train with home physical training programs. Unfortunately, the level of adherence to these programs, is often low due to lack of motivation, which is fundamental to the success of any therapeutic intervention. So, these programs need to be systematized, with objective measurements and goals in order to be more efficient [14].

A therapeutic intervention that favors balance training and adherence to home physical training programs that allows remote supervised control is tele-rehabilitation based on virtual reality (VR). VR systems for rehabilitation purposes include peripheral devices are used to transfer patient movement to the virtual environment, providing feedback on performance in a playful and motivating manner, which reinforces adherence to high-dose repetitive functional training [15–18].

VR therapy meets several criteria to be considered as an effective treatment: it provides cognitive-motor training, follows evidence-based neuroscience principles, offers motivational activities and empowerment techniques. This helps to promote self-confidence, self-management, self-efficacy and therefore independence, promoting recovery and increased quality of life for patients [19–21].

However, evidence is still scarce, heterogeneous, and inconsistent in determining inclusion criteria for balance training programs. Additionally, the researchers recommend that current VR systems should be developed considering the type of patient and the physiotherapists’ clinical criteria to facilitate high quality, effective and safe interventions that significantly improve the clinical outcomes of the patients [22–24].

Therefore, considering the implementation problems mentioned, two objectives are proposed in this study. The first is the selection of balance training with the use of virtual reality according to the clinical criteria and agreement of a group of physiotherapists dedicated to the area of neurorehabilitation and the second goal is the implementation of a clinical protocol that will be preliminarily tested in a group of patients with balance disorders belonging to an outpatient neurorehabilitation center.

### Approval

Descriptive study performed out in the outpatient neurorehabilitation area of a private clinic between July 2018 and February 2020 and was previously reviewed and approved by the Research and Clinical Trials Unit and the Ethics Committee of Clínica Alemana Universidad del Desarrollo (register number: 2019–789), Chile. With two phases the implementation. The study protocol is performed in accordance with the relevant suggestions [15, 17, 20, 25–29, 31–34].

## Methods

### Phase 1. Cluster design

#### Material

**Fig. 1:**
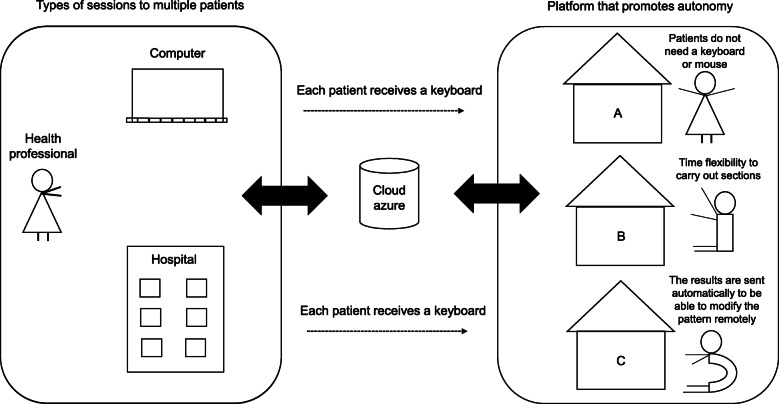
Software operation for home environment

The RehaMetrics® VR not immersive software (version 1.0, Rehametrics SL) (Fig. 1) was used, which includes more than 80 motor exercises. An Xbox One EN / XV / RF / ES AOC HD Kinect sensor is required for technical operation. An Azure Cloud server is also required (data storage based on Azure Cloud Server).

Program operation: The system uses a non-immersive virtual environment, which captures the natural movement of the patient to interact with the system and their tasks through the Kinect sensor. It combines various techniques used by the video game industry to increase patient motivation. All exercises are guided by the clinical team. They have multiple customization options. Each exercise is programmed from basic to advanced level. The level of difficulty (“difficulty”) increases as time pressure increases, visual aid decreases, increases in visual distractors, increases the base of support, among others. It collects information on the number of hits, errors, response times, height and width of the steps with respect to an axis, which as a whole constitutes the concept of “performance”, which is the result of all these parameters, including whether it was performed during the total of the programmed time. Finally, the system quantifies and stores this information and generates automatic reports, avoiding the use of paper.

#### Participants

Four physiotherapists belonging to the research area of the clinic’s Physical Medicine and Rehabilitation Service analyzed the objectives, progression and level of difficulty of each of the exercises. The physiotherapists certified with more than 3 years of experience in the care of adult neurological patients, they were divided into two groups: group 1 was called the “experts”, since they had experience in the use of the MiniBESTest (MBT) [22], programming in virtual reality and more than 3 years of experience in neurorehabilitation. Group 2 was called the “novices”, since they did not have that background.

#### Evaluation selection criteria

The clinical scales were: MBT that evaluates dynamic balance [35]; the Functional Gait Assessment (FGA) that evaluates walking stability; the Montreal Cognitive Assessment (MoCA) that evaluates the cognitive condition; and the 6 min Test (6MWT) that measures the number of meters walked in a period of time.

#### Selected exercises clusters

In Table 1, the RehaMetrics® exercises were grouped into 5 balance-related clusters. This decision was made based on an expert opinion. Clusters 1, 2 and 3 contained exercises exclusively involving displacement of the center of gravity. Clusters 4 and 5 involved displacement of the center of gravity and movement of the support base.

**Table 1:** Training clusters implemented in the study

CLUSTER 1:	CLUSTER 2:	CLUSTER 3:
1. *Antero-posterior trunk control 2. *Sitting- standing transfer 1 3. Lateral trunk control 1 4. Lateral trunk control 2 5. Trunk control 1 6. Trunk control 2	1. *Sitting- standing transfer 2 2. *Static balance 1 3. Lateral static balance 4. Antero- posterior static balance 5. Static balance 1 6. Static balance 3	1. *Simultaneous coordination 1 2.*Alternating coordination 1 3. Dynamic balance 1 3. Dynamic balance 2 4. Dynamic static balance 6. Dynamic balance 3 7. Static coordination 1 8. Static coordination 2 9. Simultaneous coordination 2 10. Alternating coordination 2
**CLUSTER 4:**	**CLUSTER 5:**	*Main exercises per cluster
1. *Side displacements 1 2. *Side displacements 4 3. Side displacements 2 4. Side displacements 3	1. *Monopodal balance 2. *Gait 3. Lower limb resistance 4. Monopodal balance with step	

**Fig. 2:**
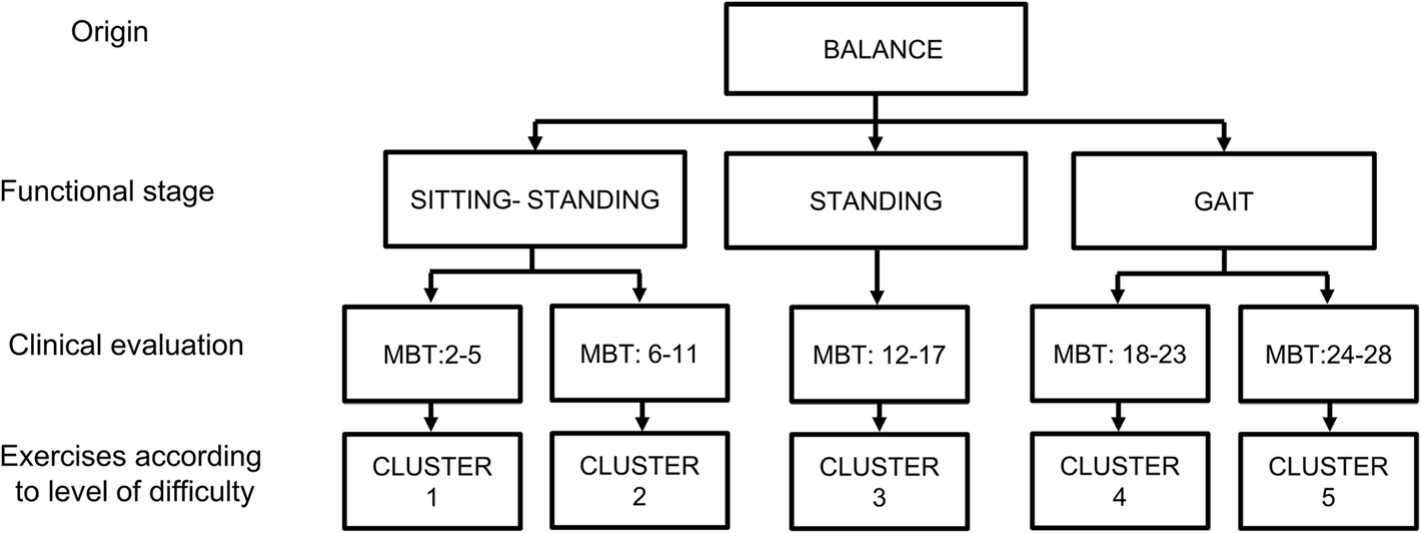
Last selection of clusters

In Fig. 2, five clusters are displayed.

- Cluster 1 included trunk control exercises conducted in a sedentary environment and a sitting-to-standing transfer exercise.
- Cluster 2 included bipedal trunk control exercises and an advanced sitting-to-standing transfer exercise.
- Cluster 3 included bipedal balance exercises and bipedal coordination exercises.
- Cluster 4 included dynamic balance exercises with changes in the lateral support base.
- Cluster 5 included walk and balance exercises with multidirectional support base changes.

#### Interobserver reliability

To evaluate the level of agreement among physiotherapists in the selection of the exercise clusters developed, 50 Random Fictional Problem Solving (RFPS) situations of patients with impaired balance for each therapist were analyzed, containing the following variables: patient’s age, diagnosis, MBT, FGA, MoCA and 6MWT scores. Ten RFPSs were designed for each of the five clusters. For the design of these cases, there was no patient participation or exact transcription of their clinical information, so in this case, informed consent was not required (the Research and Clinical Trials Unit and the Ethics Committee of the Clinica Alemana Universidad del Desarrollo, Chile (register number: 2019–789) waive informed consent), In order to include the widest range of MBT scores, two face-to-face meetings were held to evaluate interobserver reliability.

#### Statistical analysis

Interobserver reliability was evaluated using Kappa for the total sample and by cluster based on the following qualitative interpretation: values < 0 indicating no agreement, 0–0.20 slight, 0.21–0.40 fair, 0.41–0.60 moderate, 0.61–0.80 substantial, and 0.81–1 almost perfect agreement [33]. The statistical software package Stata 15 (College Station, Texas 77,845, USA) was used for all statistical analyses.

### Phase 2. Protocol implementation

#### Materials

It requires the patient to have a Smart TV at home, a solid Wi-Fi network, and a physical space 3 m long by 2 m wide. The clinical center rents the patient a NUC (Next computing Unit) 7i7 bnh computer. An Xbox One EN / XV / RF / ES AOC HD Kinect sensor a kinect. One Logitech K400 Plus 2.4Ghz Wireless Keyboard 920–007123. All these items are returned at the end of the 4 weeks of training. Teleconferences (face-to-face meetings) are held through a video conferencing program.

#### Patients

16 were recruited patients were recruited in total, 6 patients with stroke, 4 patients with idiopathic Parkinson’s disease (IPD), 1 patient with neurodegenerative disease, 1 patient with Traumatic brain injury (TBI), and 5 patients with vestibular syndrome (VS). The patients were recruited from April 2019 to February 2020. The inclusion criteria were: sedentary trunk control (Berg Balance Scale = 3 points in item 3), mild or normal cognitive level (Montreal Cognitive Assessment > 21 points), and having been prescribed telerehabilitation by a physician. Also, the patient must have a caregiver or companion to supervise their therapy while it is being performed.

#### Duration of the program

**Fig. 3:**
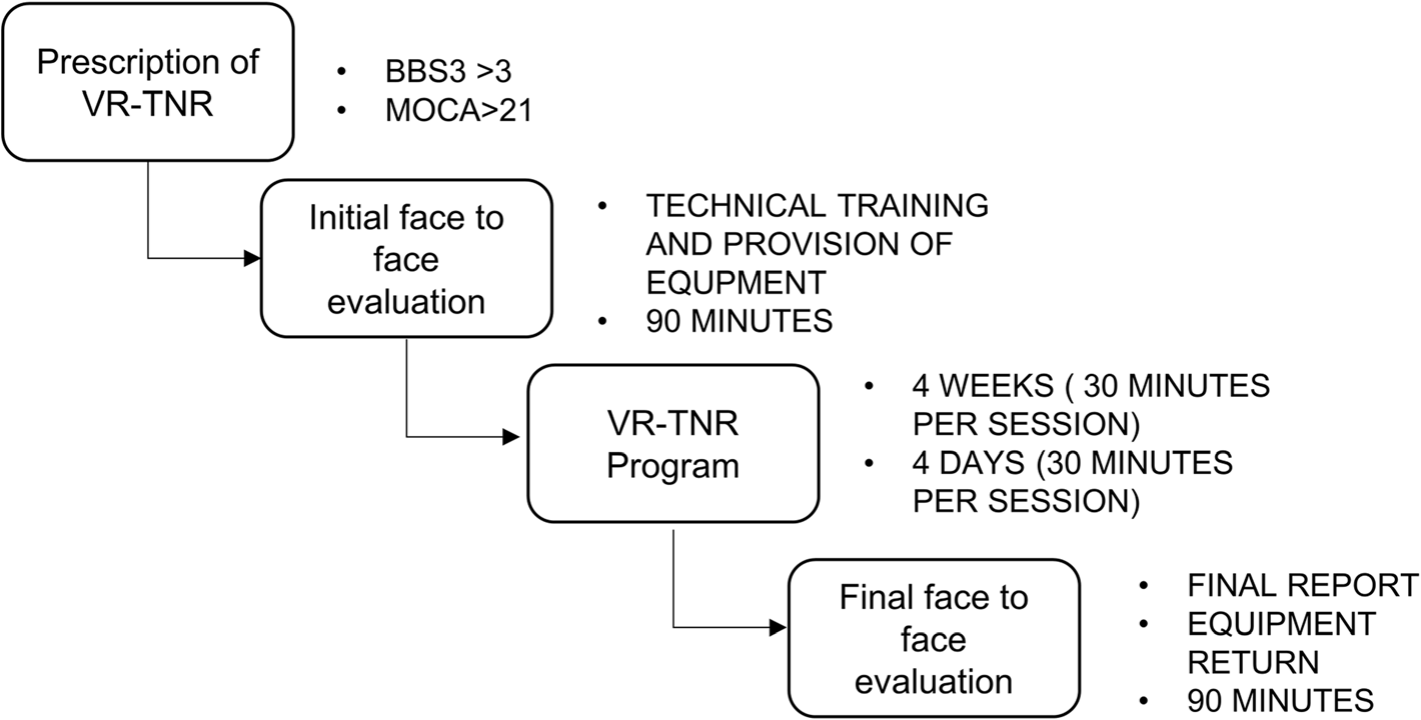
Virtual Reality Tele-neurorehabilitation protocol

4 weeks, it contemplates an evaluation at the beginning and at the end of the intervention, and it is divided into 4 stages. In Fig. 3, this protocol is displayed.

- S1: Medical indication of Teleneurorehabilitation based on Virtual Reality (VR-TNR). The patient is assessed to see whether he or she meets the inclusion criteria and if he or she has a Smart TV, a solid Wi-Fi network and physical space to train.
- S2: Face-to-face assessment at the clinical center: The patient is assessed by the physiotherapist, who uses 4 clinical scales: MiniBEST (MBT), Functional Gait Assessment (FGA), Montreal Cognitive Assessment (MoCA) and 6-Minute Test (6MWT). Technical training is also provided to instruct on the operation of the equipment. An Informed Consent is signed to participate in the study.
- S3: The patient begins training at home, in schedules chosen by him, except one day a week, when a synchronous session is performed through the video conferencing program. Virtual space is rented by the clinic, so that each session is recorded for the patient’s ethical and legal protection.
- S4: Face-to-face assessment in the clinical center that includes the application of the 4 clinical scales. The main data obtained from the software are reviewed, such as: the direction of the gravity center, in the case of static balance training, and the height and width of the step in the case of dynamic balance training. Information about the adherence to the program is obtained, the minutes of training is recorded, and the respective report is prepared. The patient completes a satisfaction survey that evaluates aspects by means of a Likert scale with scores between 1 and 5 that reflect greater or lesser satisfaction. Finally, the need to continue another cycle or finish the training is determined in conjunction with the treating physician.

#### Statistical analysis

Data are presented as means and standard deviations or medians and interquartile ranges, according to the distribution of quantitative variables, and absolute and relative frequencies for qualitative variables. The different measurements were analyzed at both points in time and compared with the Wilcoxon test for paired data. They were explored the differences between repeated measurements for the total sample and per diagnostic group, they were checked whether the differences were significant with student t-test and then verified whether the differences were significant per diagnostic group through Anova test for all statistical analyses, the statistical software package Stata 15 (College Station, Texas 77,845, USA) was used.

## Results

### Phase 1. Cluster design

The percentage of agreement among the experts irrespective of the cluster exceeded 80%, which indicates a very good strength of agreement, while the novices reached a level of agreement of 45%, which suggests a moderate strength of agreement. In Table 2, it shows a greater dispersion in the novice scores.

**Table 2:** Cohen’s kappa coefficient by type of physiotherapist and cluster

Kappa	Experts	Novices
Cluster 1	0.37	0.08
Cluster 2	0.74*	−0.20
Cluster 3	0.54*	−0.16
Cluster 4	0.43*	0.13
Cluster 5	–	−0.08
Total	0.82*	0.45*

*p value < 0.05

### Phase 2. Protocol implementation

A total of 16 patients were included in the study, the average age was 60 years old (SD 21.1 years), 62% (*n* = 10) were male and the diagnostic distribution was mainly for Stroke (37%) and Vestibular disorders (31%). 12.5% (*n* = 2) reported living in intermediate cities and 56.3% were classified as acute patients (less than 3 months of evolution).

Both the rehabilitation parameters and the clinical outcomes showed an increase in the median value in the second evaluation moment, however, only the maximum width of the left side (*p* value = 0.009), the median of the MiniBESTest score (*p* value = 0.006) and Montreal Cognitive Assessment (*p*-value = 0.020) were statistically significant.

As the Cluster is greater, the training time decreases. This occurs because the software has fewer exercises with changes in the base of support. The general performance was greater than 80%. Difficulty level increased almost 6 times within 4 weeks of training. In general, the patients were admitted with a good level of balance (MiniBestest> 24 pts), therefore the training was programmed mainly based on exercises that included changes in the base of support Table 3.

**Table 3:** Measurements of software data and clinical outcomes before and after training with VR-TNR program

	Time 1 ***n*** = 16 Median (IR)	Time 2 ***n*** = 16 Median (IR)	Value ***p***
**GENERAL TRAINING INFORMATION**			
Performance (%)		89.6 (81.3; 94.7)	
Data evaluation (days)		27.5 (24.5; 29.5)	
Training time (minutes) per cluster			
Cluster # 3 (*n* = 2)		519 (280–757)	
Cluster # 4 (*n* = 1)		509 (509–509)	
Cluster # 5 (*n* = 13)		434 (267–668)	
Difficulty	12.5 (8.9; 31.4)	71.5 (46.4; 88.8)	< 0.001
**REHAMETRICS DATA**			
Maximum Height Right Side (MHRS) (cm)	14.0 (0; 32.5)	23.5 (0; 29.0)	0.917
Maximum Height Left Side (MHLS) (cm)	19.5 (0; 31.0)	25.0 (4.5; 34.5)	0.347
Maximum Width Right side (MWRS) (cm)	20.0 (0; 26.0)	28.5 (10.0; 37.0)	0.155
Maximum Width Left Side (MWLS) (cm)	20.5 (1.5; 24.0)	28.0 (9.5; 36.0)	0.009
**CLINICAL OUTCOMES**			
MiniBESTest (MBT)	24.5 (15.0; 26.0)	26.0 (23.0; 28.0)	0.006
Functional Gait Assessment (FGA)	26.5 (19.5; 30.0)	28.0 (25.0; 29.0)	0.045
Montreal Cognitive Assessment (MOCA)	24.0 (9.5; 26.5)	25.0 (21.0; 28.5)	0.020
6 Minutes Walk Test (6MWT)	402 (352; 520)	450 (379; 570)	0.105

Performance, days of evaluation and training time per cluster are only measured at the end

The median time of evolution in months was 3 (R.I. 2–36 months). All clinical outcomes showed statistically significant differences between the median times, as did the Maximum Width Left Side (MWLS) (cm). No delta was statistically significant independent of the diagnoses, the training times in days were similar, except in the patient with TBI, who started in Cluster 3. Patients with Stroke and vestibular disorders began their training in Cluster 5, therefore they had a good level balance, reaching an optimal level at the end of the training (MiniBestest = 28 points). In contrast, patients with Parkinson’s disease began their training in lower clusters [3]. The latter is evidenced in the − 14 score in the changes produced in the 6 Minutes Walk Test Table 4.

**Table 4:** Changes of software data y clinical outcomes per diagnostic group

	Vestibular disorders ***n*** = 5 Median (IR)	Stroke ***n*** = 6 Median (IR)	Parkinson’s disease and Movement disorders ***n*** = 4 Median (IR)	Brain Trauma Injury ***n*** = 1 Median (IR)	Value ***p***
**GENERAL TRAINING INFORMATION**					
Performance (%)	92.2 (90.4; 95.3)	82.7 (78.7; 90.1)	90.6 (83.8; 96.1)	72.7 (72.7; 72.7)	0.172
Data evaluation (days)	29 (24; 31)	27 (26; 28)	30 (20; 32)	12 (12; 12)	0.435
Training time (minutes) per cluster n (%)					
Cluster # 3			757 (757–757)	280 (280–280)	0.317
Cluster # 4			509 (509–509)		
Cluster # 5	374 (337–574)	607 (434–806)	174 (131–217)		0.085
Difficulty	61.9 (41.7; 70.8)	59.8 (35.3; 78.2)	28.2 (18.2; 41.1)	37.8 (37.8; 37.8)	0.485
**REHAMETRICS DATA**					
Maximum Height Right Side (MHRS) (cm)	−3 (−15; 2)	−3 (−16; 15)	1 (0; 15.5)	0 (0; 0)	0.795
Maximum Height Left Side (MHLS) (cm)	7 (−4; 11)	6 (−8; 32)	0 (−2; 2)	0 (0; 0)	0.927
Maximum Width Right side (MWRS) (cm)	18 (2; 21)	10 (−3; 21)	0 (−1; 0)	0 (0; 0)	0.586
Maximum Width Left Side (MWLS) (cm)	17 (11; 19)	11 (6; 22)	0 (−2; 1)	0 (0; 0)	0.099
**CLINICAL OUTCOMES**					
MiniBESTest (MBT)	2 (0; 5)	3 (2; 3)	1 (−2; 3)	1 (1; 1)	0.650
Functional Gait Assessment (FGA)	0 (0; 3)	2 (0; 3)	1 (−2; 4)	2 (2; 2)	0.973
Montreal Cognitive Assessment (MOCA)	4 (0; 4)	1 (0; 3)	2 (1; 3)	0 (0; 0)	0.463
6 Minutes Walk Test (6MWT)	40 (0; 65)	46 (0; 60)	−14 (−65; 0)	61 (61; 61)	0.152

Performance, evaluation days and cluster are not expressed as changes because they are only measured at the end

**Fig. 4:**
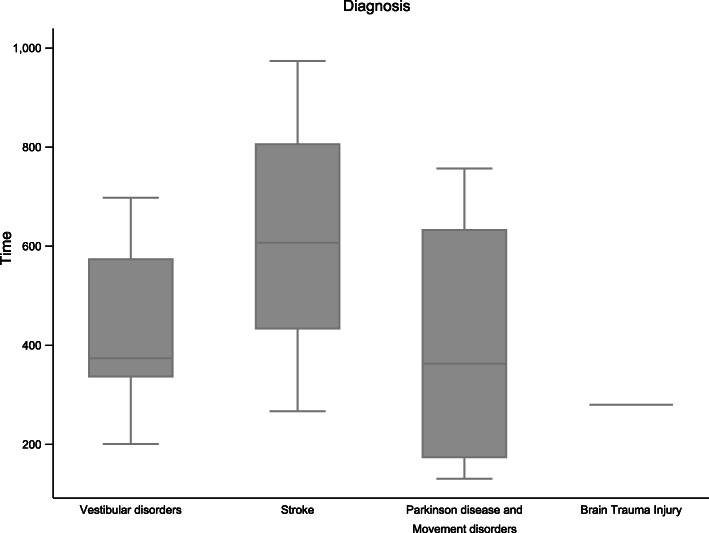
Adherence time according to diagnosis

With regard to adherence, one advantage of VR-TNR over CT is monitoring with respect to the number of work sessions performed by the patients and the duration of each of the sessions. The average number of minutes of training was 485.81 min (SD 246.49 min), and the number of sessions performed during the 4 weeks of intervention was 17 sessions (SD 7.15 sessions) (Fig. 4).

The satisfaction survey revealed that all patients scored 1 in questions 3 to 7, indicating that they were extremely satisfied. In question 8 (VR-TNR is better than Conventional Therapy (CT)) and 9 (Right Price) 50% of the patients were divided between score 2 and 3. Suggesting that they were very satisfied with these aspects. Finally, question 10 (Recommend this therapy to others) was rated by 81% (*n* = 13) with a score of 1, which means that they would recommend it. (Appendix 1) (Table 5).

**Table 5:** Median score per question of the satisfaction survey^a^

Questions	Median (Interquartile ranges)
Information about treatment	1 (1–1)
Understanding the training	1 (1–1)
Software usability	1 (1–1)
Therapeutic supervision	1 (1–1)
Satisfaction with the therapy	1 (1–1)
VR-TNR is better than Conventional Therapy (CT)	1 (1–2)
Right price	1 (1–2)
Recommending this therapy to others	1 (1–1)

^a^2 of the questions are omitte because they correspond to the age range and gender that are already exposed at the beginning of the results

(1 means that you are satisfied with the service provided and 5 means that you are dissatisfied with the service provided)

## Discussion

The results of the first phase show a highly challenging theoretical basis, such as the design of exercise clusters for patients with balance disorders developed in this analysis what had excellent interobserver reliability with trained physiotherapists. Future studies should implement these exercise clusters in real patients to identify their feasibility, safety, and clinical usefulness.

Regarding the second phase of the study, the results show a statistically significant difference between the initial and final evaluation of the clinical tests, which could result in better performance in aspects such as: balance, gait functionality, meter walked and cognition. The process of choosing adequate clinical scales is relevant, not necessarily associated with clinical diagnosis, but rather with functional diagnosis. Most patients with neurological or vestibular syndromes will fail in balance and gait stability tests, therefore, it is necessary that physiotherapists choose the most appropriate treatment at the level of balance presented by the patient, preventing the rise over assessment of his condition. Balance is often worked with a lower level of difficulty when the patient presents better levels.

Regarding the incorporation of clusters, it is possible to relate the adequate selection of the cluster, which considers the initial individual balance level to the high performance (greater than 80%). In relation to differential diagnoses, it is possible to observe in patients with Parkinson’s disease a stagnation in cluster 3, which reflects the difficulty to achieve lateral movements and gait. This could suggest that this group of patients requires longer programs, or an annual dosage, where they perform this training a couple of times a year in order to maintain their balance and gait skills [36, 37].

Patients evaluated and treated showed significant reduction in center-of-mass displacement. The abnormal postural responses observed in these patients might contribute to the well-known age-related difficulty in dealing with balance control [2].

The physiotherapist’s role changes when implementing telerehabilitation, meaning that they must use new information and must be prepared (“readiness”) to efficiently transform it according to their clinical reasoning. This transformation of information from virtual reality programs to the clinical language of physiotherapists is fundamental for the usability of these systems [22, 23].

One way of carrying out this process is the clinical analysis of the VR programs, to adapt them to the clinical reality of the physiotherapists who will use them. To this end, it was proposed grouping the information in exercise clusters, defined by the same physiotherapists using common eligibility criteria in their daily practice [38].

It is necessary that the training criteria are related to the clinical evaluation of balance and that training is based on the functional level of the patient, considering the progression in speed to change from one position to another, in the ability to perform monopodal support and possibility to shift the center of gravity in different directions, all this is feasible with this virtual reality program [12].

Preliminary evidence suggests to determine whether reactive stepping characteristics (frequencies of extra steps, lateral steps, foot collisions) and timing (foot- off time, swing time) in response to support-surface perturbations improved following 6-weeks of training [12]. This study proposed a period of 1 month, because not all patients start their treatment in more advanced stages, however for this group the recommendation could be to extend the training period.

Regarding high adherence, the evidence suggests [15, 17, 20, 25–29, 31–34] that it is related to the following factors: practicing a relatively simple, low-cost program, exercising at least twice a week, with monitoring and assessment at least once monthly. In addition, it can provide a useful motivational strategy. Therefore, this study focused on following these recommendations for the search for better adherence, therefore, specific objectives were sought, training times were limited, as well as contact times with the therapist, whose function is essential to motivate the progress of the patient.

In any case, the weakness of this study is the scant evidence to find a classification process such as the cluster design, therefore it must be tested in a larger population if this proposed design is really effective to establish a parameter for future studies. Another limitation was the variability of clinical diagnosis and the sample showed. Although this program has many attributes, such as the information it provides to the patient and the clinical team, in terms of level of difficulty, performance, adherence to the program, among others, it requires further analysis to objectively interpret the results, since Its interpretation depends on the correct choice of exercise clusters, on the one hand, and its scientific dissemination is limited, because each program is different and different parameters are measured, increasing variability. Therefore, the challenge in this area is to improve decision making in rehabilitation practice, achieve unification of evaluation criteria, efficient dosage of training, and education of each rehabilitation team. For future studies, follow-up evaluation is necessary to check the clinical evolution, including a large and specific sample size, however, for the little evidence in our environment, this study represents a first step in the advancement and knowledge of these challenging and unexplored training strategies.

The Strengths of this study are in 3 components: The selection of clinical scales associated with impaired balance is crucial to determine the treatment to be followed. Then, the treatment should be oriented to train the potential areas, considering the relevant components, such as reactive movement strategies and cognitive processing, which are not always included in the evaluation and treatment of balance impairment. In addition, it has been suggested that the balance is according to the subsystems involved and not necessarily according to the pathology [39]. Despite the fact that this VR program comes with a predetermined group of exercises, there may be a risk of low usability by physiotherapists due to the variability in knowledge or clinical experience in this type of treatment. That is why it was found necessary that expert physiotherapists analyze the virtual platform so that the program has an efficient usability. Although the gamification of balance exercises in clusters has no evidence, the studies do not necessarily detail the logic and dosage of training, so it is believed that it is necessary to deepen the analysis of the training provided in order to understand the mechanisms involved in the recovery of balance [40]. Finally, the proposal of a protocol allows to systematize clinical care, measure the effect in terms of quality, efficiency, and determine the clinical conduct to follow [31].

## Conclusions

VR-TNR is an excellent alternative to provide continuity of treatment to patients, which will probably result in a more widespread use. Also, it allows to improve decision making in rehabilitation practice. The inclusion criterion is fundamental. Patients with different levels of balance impairment could improve their clinical outcomes with constant training and go to the next level.

Finally, this program allows the therapist, therefore, the clinical center, to keep an objective record of the patient’s clinical progress and, at the end, to contrast the information of his progress with the clinical evaluations, optimizing the clinical process in its entirety.

## Supplementary Information

**Additional file 1.**

## Data Availability

The datasets used and/or analysed during the current study are available from the corresponding author on reasonable request.

## Abbreviations

VR-TNR: Teleneurorehabilitation based on Virtual Reality.
MBT: MiniBESTest
FGA: Functional Gait Assessment
MoCA: Montreal Cognitive Assessment
6MWT: 6 min Test
RFPS: Random Fictional Problem Solving
NUC: Next computing Unit
IPD: Idiopathic Parkinson’s disease
TBI: Traumatic brain injury
VS: Vestibular syndrome
MWLS: Maximum Width Left Side
CT: Conventional Therapy
