# Supplementary material for: Teleneurorehabilitation program (virtual reality) for patients with balance disorders: descriptive study
Source: BMC Sports Sci Med Rehabil. 2021 Aug 2;13:83. doi: 10.1186/s13102-021-00314-z (PMC8330090; doi:10.1186/s13102-021-00314-z)
Supplement: Supplementary file 1 — Additional file 1. [file 13102_2021_314_MOESM1_ESM.docx]

Appendix 1

| 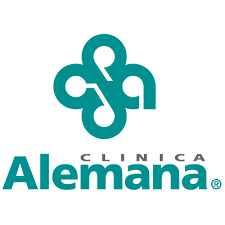 | **Satisfaction survey** |
| --- | --- |

The Physical Medicine and Rehabilitation Service of Clinica Alemana invites you to answer the following user satisfaction survey.

Please mark your answer with an X. 1 means that you are satisfied with the service provided and 5 means that you are dissatisfied with the service provided. Your response will help improve our service to provide you with a better experience.

Thank you very much for participating.

| 1. What is your age group? | 17 or less | 18-20 | | 21-29 | | 30-39 | | 40-49 | | 50-59 | | 60 or more |
| --- | --- | --- | --- | --- | --- | --- | --- | --- | --- | --- | --- | --- |
|  |  | | | | | | | | | | | |
| 2. What is your gender? | Man | | | | | | Woman | | | | | |
|  |  | | | | | | | | | | | |
| 3. How informed are you about the goals of therapy? | 1  Extremely informed | | 2  Very informed | | 3  Moderately informed | | | | 4  A little informed | | 5  Nothing informed | |
|  |  | | | | | | | | | | | |
| 4. Overall, how clear was the training you received? | 1  Extremely clear | | 2  Very clear | | 3  Moderately clear | | | | 4  Unclear | | 5  Nothing clear | |
|  |  | | | | | | | | | | | |
| 5. How satisfied are you with the ease of use of this software? | 1  Extremely satisfied | | 2  Very satisfied | | 3  Somewhat satisfied | | | | 4  Not so satisfied | | 5  Not satisfied at all | |
|  |  | | | | | | | | | | | |
| 6. In general, how well did you find the therapeutic assistance? | 1  Extremely good | | 2  Very good | | 3  Something good | | | | 4  Not so good | | 5  Nothing good | |
|  |  | | | | | | | | | | | |
| 7. Overall, are you satisfied or dissatisfied with the experience of this therapy? | 1  Very satisfied | | 2  Satisfied | | 3  Neither satisfied nor dissatisfied | | | | 4  Dissatisfied | | 5  Very dissatisfied | |
|  |  | | | | | | | | | | | |
| 8- Compared to conventional therapy, do you consider telerehabilitation to be better, worse or almost the same? | 1  Better | | 2  Something better | | 3  Almost equal | | | | 4  Something worse | | 5  Much worse | |
|  |  | | | | | | | | | | | |
| 9. Would you say this type of therapy is worth what it cost? | 1  Definitely yes | | 2  To a certain extent yes | | 3  Neither one nor the other | | | | 4  To some extent no | | 5  Definitely not | |
|  |  | | | | | | | | | | | |
| 10. In general, what is the probability that you recommend this type of therapy? | 1  Extremely likely | | 2  Quite likely | | 3  Somewhat probable | | | | 4  Unlikely | | 5  Not likely | |
